# Standardizing Hyperthermic Intraperitoneal Chemotherapy in Ovarian Cancer Treatment: Navigating Complexities and Charting the Path Forward

**DOI:** 10.3390/cancers16020400

**Published:** 2024-01-17

**Authors:** Juan José Segura-Sampedro, Pedro Cascales-Campos

**Affiliations:** 1 General & Digestive Surgery Service, La Paz University Hospital, 28046 Madrid, Spain; 2 School of Medicine, University of the Balearic Islands, 07122 Palma de Mallorca, Spain; 3 Health Research Institute of the Balearic Islands, 07122 Palma de Mallorca, Spain; 4 Department of General and Digestive Surgery, Virgen de la Arrixaca University Hospital, IMIB-Arrixaca, 30120 Murcia, Spain; cascalescirugia@gmail.com; 5 Digestive and Endocrine Surgery and Transplantation of Abdominal Organs Research Group, Biomedical Research Institute of Murcia (IMIB), 30120 Murcia, Spain

**Keywords:** HIPEC, cytoreductive surgery, ovarian cancer

The incorporation of hyperthermic intraperitoneal chemotherapy (HIPEC) into the treatment landscape for ovarian cancer has invoked a spectrum of emotions, ranging from enthusiastic anticipation to cautious skepticism. The ongoing exploration of HIPEC’s application in managing advanced ovarian cancer represents a pivotal juncture, with the potential to redefine established treatment paradigms. However, this transformative journey is accompanied by a myriad of complexities, nuances, challenges, and potential trajectories, underscoring the dynamic evolution of ovarian cancer therapeutics.

The discourse surrounding using HIPEC to treat ovarian cancer is multifaceted. Beyond the initial excitement, a rich tapestry of debates has unfolded [1], delving into questions about its efficacy, the criteria for patient selection, and its impact on overall survival. In an effort to enhance the treatment of ovarian cancer, particularly in the peritoneal cavity—the primary site of disease spread—intraperitoneal chemotherapy and HIPEC have emerged. Recent studies indicate that HIPEC can provide a survival advantage in certain scenarios in the treatment of ovarian cancer [2]. Notably, this benefit comes without the introduction of additional concerns such as heightened morbidity, mortality, or a compromise in the quality of life. Despite these positive findings, several questions still linger regarding the best practices for utilizing HIPEC. These uncertainties include considerations about the most effective agents, appropriate dosage levels, and the ideal temperatures for its application. The lack of standardization and the heterogeneity in dosages and durations emerge as formidable hurdles, as exemplified by the study by Cascales et al. [3], which sheds light on this major challenge facing HIPEC.

In response to these challenges, significant efforts are underway. The study by Acs et al. [4], comparing HIPEC application times of 60 and 90 min, with superior results at longer exposure times, seeks to contribute valuable evidence toward standardizing HIPEC application in Advanced Primary Epithelial Ovarian, Fallopian Tube, and Primary Peritoneal Cancer. Additionally, the work of Horvath et al. [5] corroborates the validity of 90 min HIPEC with cisplatin as monotherapy, specifically in the case of Peritoneal Recurrence of Platinum-Sensitive Recurrent Ovarian Cancer. Furthermore, this Special Issue’s final papers [6,7] highlight the survival benefit of HIPEC in patients with Advanced Epithelial Ovarian Cancer undergoing interval cytoreductive surgery.

While these studies primarily focus on the application of HIPEC, they collectively emphasize the need to redefine the longstanding goal of developing an optimal cytoreductive surgery method [8]. The complete eradication of visible disease has emerged as a pivotal factor, which is partially responsible for the favorable outcomes that have been observed in groups performing complete cytoreduction associated with HIPEC.

As we peer into the future of HIPEC, it is evident that ongoing and planned studies worldwide are dissecting its potential in frontline scenarios and exploring non-cisplatin regimens [9]. Amidst the persisting uncertainties, a promising future unfolds. A common call resonates, emphasizing the imperative for meticulous trial design and efficient accrual to ensure the relevance of results in this rapidly evolving landscape. The collective endeavor to unlock the true potential of HIPEC is underway, promising to reshape the narrative of ovarian cancer therapeutics.

## References

[1] Harter P., Bogner G., Chiva L., Cibula D., Concin N., Fotopoulou C., Gonzalez-Martin A., Guyon F., Heinzelmann-Schwarz V., Kridelka F. (2023). Statement of the AGO Kommission Ovar, AGO Study Group, NOGGO, AGO Austria, Swiss AGO, BGOG, CEEGOG, GEICO, and SFOG Regarding the Use of Hyperthermic Intraperitoneal Chemotherapy (HIPEC) in Epithelial Ovarian Cancer. Bull. Cancer.

[2] Gelissen J.H., Adjei N.N., McNamara B., Mutlu L., Harold J.A., Clark M., Altwerger G., Dottino P.R., Huang G.S., Santin A.D. (2023). Hyperthermic Intraperitoneal Chemotherapy in Ovarian Cancer. Ann. Surg. Oncol..

[3] Gil A.G., Palencia C.F.-D., Ruiz J.G., Gil Gómez E., Hernández F.L., Ruiz A.N., Martínez J., Marhuenda I., Campos P.A.C. (2023). HIPEC in Ovarian Cancer Is the Future… and Always Will Be? Results from a Spanish Multicentric Survey. Cancers.

[4] Acs M., Herold Z., Szasz A.M., Mayr M., Häusler S., Piso P. (2022). Prolonged Exposition with Hyperthermic Intraperitoneal Chemotherapy (HIPEC) May Provide Survival Benefit after Cytoreductive Surgery (CRS) in Advanced Primary Epithelial Ovarian, Fallopian Tube, and Primary Peritoneal Cancer. Cancers.

[5] Acs M., Gerken M., Schmitt V., Piso P., Königsrainer A., Baransi S., Yurttas C., Häusler S., Horvath P. (2023). Role of HIPEC after Complete Cytoreductive Surgery (CRS) in Peritoneal Recurrence of Platinum-Sensitive Recurrent Ovarian Cancer (OC): The Aim for Standardization at Two Reference Centers for CRS. Cancers.

[6] Llueca A., Ibañez M.V., Cascales P., Gil-Moreno A., Bebia V., Ponce J., Fernandez S., Arjona-Sanchez A., Muruzabal J.C., Veiga N. (2023). Neoadjuvant Chemotherapy plus Interval Cytoreductive Surgery with or without Hyperthermic Intraperitoneal Chemotherapy (NIHIPEC) in the Treatment of Advanced Ovarian Cancer: A Multicentric Propensity Score Study. Cancers.

[7] Aronson S.L., Lopez-Yurda M., Koole S.N., Schagen van Leeuwen J.H., Schreuder H.W.R., Hermans R.H.M., de Hingh I.H.J.T., van Gent M.D.J.M., Arts H.J.G., van Ham M.A.P.C. (2023). Cytoreductive Surgery with or without Hyperthermic Intraperitoneal Chemotherapy in Patients with Advanced Ovarian Cancer (OVHIPEC-1): Final Survival Analysis of a Randomised, Controlled, Phase 3 Trial. Lancet Oncol..

[8] Segura-Sampedro J.J., Morales-Soriano R., Arjona-Sánchez Á., Cascales-Campos P. (2020). Secondary Surgical Cytoreduction Needs to Be Assessed Taking into Account Surgical Technique, Completeness of Cytoreduction, and Extent of Disease. World J. Surg. Oncol..

[9] Ukegjini K., Guidi M., Lehmann K., Süveg K., Putora P.M., Cihoric N., Steffen T. (2023). Current Research and Development in Hyperthermic Intraperitoneal Chemotherapy (HIPEC)—A Cross-Sectional Analysis of Clinical Trials Registered on ClinicalTrials.Gov. Cancers.

